# Tumor Microenvironment: A New Treatment Target for Cancer

**DOI:** 10.1155/2014/351959

**Published:** 2014-04-10

**Authors:** Ming-Ju Tsai, Wei-An Chang, Ming-Shyan Huang, Po-Lin Kuo

**Affiliations:** ^1^Division of Pulmonary and Critical Care Medicine, Department of Internal Medicine, Kaohsiung Medical University Hospital, Kaohsiung Medical University, Kaohsiung, Taiwan; ^2^Graduate Institute of Medicine, College of Medicine, Kaohsiung Medical University, Kaohsiung, Taiwan; ^3^Institute of Clinical Medicine, College of Medicine, Kaohsiung Medical University, Kaohsiung, Taiwan; ^4^Department of Internal Medicine, School of Medicine, College of Medicine, Kaohsiung Medical University, Kaohsiung, Taiwan; ^5^Department of Medical Research, Kaohsiung Medical University Hospital, Kaohsiung Medical University, Kaohsiung, Taiwan; ^6^Institute of Medical Science and Technology, National Sun Yat-Sen University, Kaohsiung, Taiwan

## Abstract

Recent advances in cancer therapy encounter a bottleneck. Relapsing/recurrent disease almost always developed eventually with resistance to the initially effective drugs. Tumor microenvironment has been gradually recognized as a key contributor for cancer progression, epithelial-mesenchymal transition of the cancer cells, angiogenesis, cancer metastasis, and development of drug resistance, while dysregulated immune responses and interactions between various components in the microenvironment all play important roles. Future development of anticancer treatment should take tumor microenvironment into consideration. Besides, we also discuss the limitations of current pre-clinical testing models that mainly come from the impossibility in simulating all detailed carcinogenic mechanisms in human, especially failure to create the same tumor microenvironment. With the cumulating knowledge about tumor microenvironment, the design of a novel anticancer therapy may be facilitated and may have better chance for success in cancer eradication.

## 1. Introduction

Cancer is a multifactorial disease and is one of the leading causes of death worldwide. The contributing factors include specific genetic background, long-term exposure to various environmental stresses, and bias diet habit. All these risk factors finally reflect on the accumulation of molecular changes in cells, which contributes to the initiation of carcinogenesis. Since some important mutated proteins, such as epithelial growth factor receptor (EGFR), p53, and c-myc, have been recognized as important contributors for carcinogenesis, they have been increasingly taken as major targets for drug development in order to eliminate mutated cancer cells [1–3]. Although this common strategy can usually achieve significant effect initially, drug resistance usually comes along with relapsing disease sooner or later. This implies some missing links between the actual underlying carcinogenic mechanisms and current drug development strategies. Tumor microenvironment may be a crucial part for these missing links.

**Figure 1:**
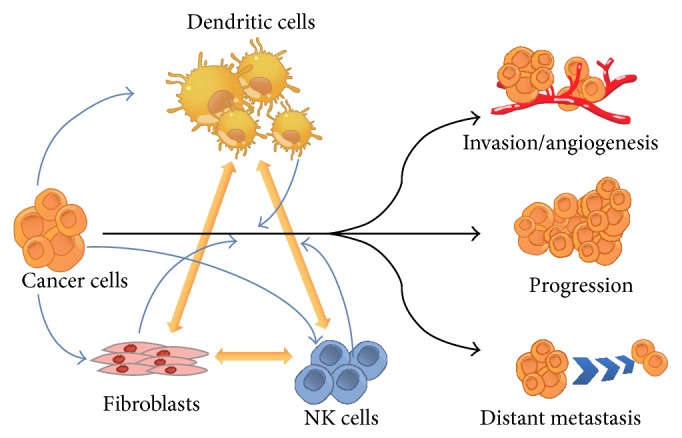
Cell-cell interactions in the tumor microenvironment contribute to cancer cell progression, invasion, and metastasis.

Recently, tumor microenvironment has been gradually recognized as a key contributor for cancer progression and drug resistance (Figure 1) [4]. This concept implies that cancer is no longer an isolated cellular population; instead, it is the consequence of collaboration of different types of malcontrolled cells. In fact, as early as 1880s, Steven Paget proposed the “seed and soil” hypothesis, suggesting that a fertile “soil” (the microenvironment) is essential for the “seed” (the tumor cells) to grow [5–7].

In this review, we summarize some important concepts of tumor microenvironment and discuss the potential clinical implications. As the microenvironment is quite complicated, we would like to focus on the role of dysregulated immune responses and interactions between various components in the microenvironment in tumor progression, invasiveness, and even development of drug resistance. In addition, this review also discusses the current preclinical testing models and highlights their unsatisfying design, suggesting the need for some new strategies in future anticancer drug development.

## 2. Dysregulated Immune Responses in Tumor Progression

A large number of clinical survey have showed that chronic inflammation is an important risk factor for tumor formation [8, 9]. These findings that directly support immune mechanisms can somehow promote tumor progression in certain condition. Indeed, chronic overexpression of inflammatory mediators is a major characteristic of tumor microenvironment and may contribute to carcinogenesis, tumor progression, and metastasis [4, 9–13]. Two types of pathways, intrinsic pathways and extrinsic pathways, lead to the formation of inflammatory microenvironment [4, 10, 14]. Intrinsically, genetic alterations within the neoplastic cells increase their production of inflammatory mediators [4, 10, 14]. Extrinsically, tumor-infiltrating cells, mainly immune cells like T cells, natural killer (NK) cells, macrophages, and dendritic cells, produce inflammatory mediators to form a microenvironment promoting cancer development and progression [4, 10, 11, 14].

Although both anticancer innate and adaptive immune responses are primitively designed for recognizing abnormal cells and further cleaning up, they usually turn into anergy state at the site of chronic inflammation [15]. The anergy state mainly results from two major causes—the gathering of immune regulatory/suppressor cells and accumulation of high concentration of immune inhibitory cytokines or associated ingredients.

Finding the existence of immune suppressor/regulatory cells is undoubtedly a great breakthrough in autoimmune and cancer research field. The immune regulatory cells come from both lymphoid and myeloid origins. The most well-known immunosuppressor of lymphoid origin is regulatory/suppressor T cells (T_reg_s), which coexpress surface markers such as CD4 and interleukin-2 receptor *α* chain (IL-2R*α*, also known as CD25), as well as a particular intracellular protein called forkhead box P3 (FoxP3) [16]. T_reg_s are usually found around tumor mass in clinical specimens and may suppress the antitumor immune responses [17]. Albeit the control mechanism is still unclear, the expression of self-peptide recognized T-cell receptor (TCR) and cytotoxic T lymphocyte-associated antigen-4 (CTLA-4, a costimulatory receptor) may play important roles [16]. T_reg_s can also secrete immune inhibitory cytokines, such as IL-10 and TGF-*β*, which can transform dendritic cells to a suppressive type and downregulate the activity of effector T cells, NK cells, and NKT cells [16].

The types of myeloid-origin suppressor cells are much diverse. A distinct subgroup of macrophage, the M2 macrophage, is important for immunosuppression [18]. Although M2 macrophage derives from monocytes and carries CD68 marker as M1 macrophage does, it can be discriminated from the M1 type by its cytokine profile and particular cell surface markers [10]. M1 macrophage expresses CCR7, while M2 macrophage expresses CD163 in dominant M2c subtype or CD206 in M2a subtype [19, 20]. In general, M1 macrophage can produce a series of proinflammatory cytokines such as IL-6, IL-12, and TNF-*α*, while M2 macrophage tends to produce immune inhibitory cytokine such as IL-10 and TGF-*β* [10, 21]. The other group of myeloid-origin suppressor cells is a diverse population named myeloid-derived suppressor cells (MDSCs), which includes granulocytes, monocytes/macrophages, and dendritic cells, depending on the types and stages of the tumor that these immune cells infiltrated [9, 18]. MDSCs present as an incompletely mature phenotype and thus may carry CD11b or CD33 markers as precursors of myeloid lineage cells do; they lack CD14 or HLA markers which are mainly expressed in mature myeloid lineage cells [9]. These cells can interfere with both innate and adaptive anticancer immunity, mainly through secreting IL-10 and downregulating IL-12 to promote Th2 dominant immune environment [9, 22, 23]. In addition, MDSCs can enhance M2 macrophage formation by cell-cell contact interaction [23, 24]. Moreover, MDSCs suppress the function of T lymphocytes, not only through expressing arginase-1 to degrade L-arginine, which decreases the expression of CD3zeta chain and cell cycle regulator in T lymphocytes, but also through producing NO, which inhibits expression of JAK3, STAT5, and MHC class II and induces T-cell apoptosis [25–30].

Based on the knowledge that the recruitment and differentiation of immune suppressor cells should be tightly regulated by cytokine or corresponsive mediators, the source of these regulatory signals ought to be questioned. The immunosuppressive nature of tumor microenvironment may be primarily attributed to the ineffective priming of the immune system against tumor-associated antigen by immunogenic signal from the tumor itself, whereas all kinds of immune cells infiltrating the tumor may participate in the process [11]. In addition to the cancer cells and immune cells, the surrounding stromal cells can also produce regulatory cytokines and mediators to participate in immune regulation [18, 31–33]. In recent years, many evidences show that the crosstalk between tumor cells and the tumor-infiltrating cells also contributes to these processes. For example, monocyte chemoattractant proteins, such as chemokine (C-C motif) ligand 2 (CCL2), secreted by many tumors mediate immunoinhibitory effects and facilitate tumor metastasis; blocking CCL2-CCR2 signalling by monoclonal antibodies has been shown to augment CD8+ T-cell-mediated responses elicited by immunotherapy and to inhibit metastatic seeding [34, 35]. CCL28 derived from tumor cells has also been shown to promote the recruitment of T_reg_s and thereby promote tolerance of tumor and angiogenesis [36]. CCL18 from tumor-associated macrophages has also been shown to promote cancer invasion and metastasis [37]. In the case of non-small cell lung cancer (NSCLC), it has been evidenced that the neoplasm and vicinal cells can release TGF-*β* or cyclooxygenase-2 (COX-2) for recruiting T_reg_s to tumor region [9]. Some inflammatory cytokines are consequent on long-term interplay of immune and cancer cells, such as prostaglandins, IL-1*β*, IL-6, and IL-13, which can trigger the expansion and activation of MDSCs [9, 38, 39]. In some cases, cancer cells can express or secrete human leukocyte antigen G (HLA-G), through which they inhibit the immune surveillance function of NK or NKT cells [40, 41]. Furthermore, our previous studies have demonstrated that lung cancer may secrete some mediators causing anergy of tumor-associated dendritic cells (TADCs) and promoting their secretion of some factors which in turn enhance cancer progression [42, 43]. By transplanting LLC adenocarcinoma cells via tail vein injection to the mice with same genetic background (C57BL/6 mice) to mimic the original lung tumor environment, we found that lung cancer cells could secrete galectin-1 to affect the differentiation of monocyte into tolerogenic dendritic cells with increased production of IL-10 [42].

## 3. Tumor Microenvironment Facilitates Tumor Invasion

The carcinogenic mutation of cells and dysregulated immune responses are just preludes for cancer progression and invasion. As suggested by the “seed and soil” concept, since mutated cells are the foundation for the malignant disease, the tumor microenvironment may be quite important in fostering the tumor cells and may substantially assist them to acquire advanced invasion ability [4–7]. Therefore, when abundant evidence showed that the epithelial-mesenchymal transition (EMT) phenomenon usually intimately correlate with chronic inflammatory situation, it is believed that certain favorable mediators which can facilitate cancer cells to evolve to much invasive type must exist in the inflammatory microenvironment around tumor mass [4, 7, 9]. Indeed, increasing evidences demonstrate that a variety of inflammatory mediators from cancer and tumor-infiltrating cells, such as IL-1, IL-6, and IL-8, facilitate the development of tumor microenvironment in favor of tumor cell proliferation, motility, invasion, and EMT and therefore increase their metastatic ability [9, 13].

EMT is a specific process by which cells with highly polarized epithelial characteristics acquire the mesenchymal trait, which is widely believed to make the cells much movable and therefore play an important role in cancer invasion and metastasis [7, 44–49]. In the cellular and molecular level, some important changes take place during EMT, including increased transcriptional repressors of E-cadherin (including Snail, Slug, Twist, and Zeb-1), E-cadherin degradation, and replacement of epithelial proteins (such as cytokeratins, apical actin-binding transmembrane protein-1, and zonula occludens-1) with mesenchymal proteins (such as vimentin and type 1 collagen) [4, 44–49]. Coincidentally, these molecular mechanisms are highly permissive in the chronic inflammation environment [50, 51]. The infiltrated immune cells can produce series of EMT-favorable cytokines, such as TGF-*β*, TNF-*α*, and IL-1*β* [4, 7, 51, 52]. The key regulatory role of TGF-*β* for EMT has been recognized in various models. It has been noticed that TGF-*β* induces EMT in alveolar epithelial cells, making them transformed to fibroblasts/myofibroblasts [52, 53]. Besides, TGF-*β* signaling elicits expression of* high mobility group A2 (HMGA2)* via Smad transducers, which then upregulates the production of Snail, Slug, and Twist and contributes to EMT [51]. TNF-*α* alone may also mediate EMT through promoting E-cadherin degradation, mainly via strengthening Snail stability in an NF-*κ*B-dependent manner [4, 54]. IL-1*β* and TGF-*β* can induce COX-2 expression, which increased prostaglandin E_2_ (PGE_2_) level, and subsequently induce EMT through the downregulation of E-cadherin via the enhanced expression of transcriptional repressors, Snail and Zeb1 [4, 55].

EMT may also be triggered in an indirect and complicated way. Our recent studies found that lung cancer cells secret galectin-1, which promotes its migration, invasion, and EMT [45]. On the other hand, our previous studies found that galectin-1 secreted by lung cancer cells may promote differentiation of monocyte to specific TADCs, which can secrete amphiregulin to enhance cancer cell proliferation, EMT, and therefore invasiveness [43]. In addition to the interactions between cancer cells and immune cells, we have also investigated the interactions between lung cancer cells and bone. We have demonstrated that lung cancer cells can not only secrete IL-8 to promote osteoclastogenesis but also trigger osteoblast to secrete bone morphogenetic protein-2 (BMP-2), which in turn promotes lung cancer migration, invasion, and EMT [48, 56].

In addition to EMT, a well-established tumor can also cooperate with adjacent stromal cells to build up highly specialized surrounding, such as vessel-rich or migration-favorable environment, facilitating further spreading out. After being influenced by abnormal paracrine signals from the tumor, the carcinoma-associated fibroblasts (CAFs) are gradually formed from the normal stromal cells through the process called stromatogenesis [31, 57]. CAFs are the main source of host-derived VEGF and may therefore contribute to angiogenesis [31, 58]. CAFs also secrete hepatocyte growth factor (HGF), which not only activate EMT-related c-Met pathway but also give lung cancer cells resistance to conventional epidermal growth factor tyrosine kinase inhibitors [7, 59].

The increased oxygen demand from uncontrolled-growing cancer and infiltrating immune cells brings about a hypoxic environment, which upregulates signal pathway dominated by hypoxia-inducible factor 1 (HIF-1) [59, 60]. The HIF-1*α* subunit, which is normally controlled by ubiquitin-mediated degradation in normoxic condition, is stabilized in hypoxic condition and further binds to HIF-1*β* chain to construct a functional heterodimer [60]. Binding of this heterodimer to hypoxia-response elements (HREs) turns on the transcription of downstream genes involved in the regulation of cell survival, proliferation, extracellular matrix remodeling, angiogenesis, and invasiveness and may therefore contribute to cancer progression [4, 60, 61]. For example, HIF-1*α*-mediated lipoxygenase pathway regulates the migration and invasion of epithelial ovarian cancer cells in hypoxic condition and promotes cancer metastasis [60]. Activation of Slug by HIF-1*α* increased the expression of membrane-type 4 matrix metalloproteinase (MT4-MMP, also known as MMP-17) in human cancer cells, which promotes* in vitro* invasiveness of the cells and* in vivo* colonization and growth of the cells in the lungs, via an EMT-independent mechanism [62]. Furthermore, the HIF pathway may also induce EMT [4]. Hypoxia or overexpression of HIF-1*α* reduces E-cadherin expression and increases cell migration, invasion, and metastasis in a Twist-dependent manner, as shown in a study using non-small cell lung cancer, human hypopharyngeal carcinoma, tongue cancer, breast cancer cell lines, and clinical specimens from head and neck squamous cell carcinoma patients [61].

## 4. Drug Resistance Related to Tumor Microenvironment

The pleiotropic nature of cytokines in the microenvironment contributes to promoting cancer cell proliferation, bypassing apoptosis, inducing EMT of cancer cells, enhancing chemokines to recruit immune suppressor cells aggregating around the tumor, and even driving the development of drug resistance. Consequently, multiple beneficial elements for tumor invasion and metastasis accumulate over time in the tumor microenvironment, which make cancer therapy much more challenging.

Many anticancer drugs have been developed for targeting the crucial signal molecules which are usually overactivated in cancerous tissue. After immune suppressive mechanism has been gradually revealed, the attempt of using drug for manipulating immune response, in terms of immunotherapy, is already on the way. However, the attempt for developing cancer-curing medications is usually frustrating because the occurrence of drug resistance seems inevitable. The concept of tumor microenvironment can provide a sort of understandable reasons for explaining how cancer finally turns the effective drug into a failure. The underpinning mechanisms are so-called “*de novo* mechanisms,” which point out that the dynamic changes of the tumor surrounding can either give the cancer cell new immortal signal or fundamentally alter some default signal pathways and thus cancer cells finally can bypass the influence caused by the original drug [63].

A new signal input which strengthens cancer cells can be given via soluble factors or physical cell-cell contact in specific tumor microenvironment. IL-6 can be exemplified as a soluble factor which deeply influence the treatment outcome in multiple myeloma models [63]. The high concentration of IL-6 usually exists in the bone marrow microenvironment to where the malignant B cells home. IL-6 transmits major survival signals through various pathways, including PI3K/AKT, Ras/Raf/MEK-ERK1/2, JAK/STAT3, SHP2/RAFTK, and Src-family tyrosine kinase pathways, and each of these pathways may give the cancer cell alternative surviving reliance other than original intrinsic mutation [63]. As for the survival signal given by the direct cell contact, integrin-mediated adhesion plays an important role. The malignant immune cells also acquire survival-related signal when their surface integrin binds to certain extracellular matrix in bone marrow sanctuary, such as fibronectin, vitronectin, laminin, and collagens, and further activates downstream associated factors [63]. This signal cascade finally modulates cytoskeleton remodeling, which then regulates cell proliferation, differentiation, and the motile ability. As shown by these examples,* de novo* mechanisms provide the rational explanation that initially functional drug may lose their targeting function after cancer cells gain more versatile survival ability after being fostered by proper microenvironment [63].

In addition to the myeloma model, the drug resistance driven by* de novo* mechanisms has been demonstrated in solid tumor models as well. The drug resistance can make either chemotherapy or antiangiogenic therapy ineffective [64]. Some studies have even shown that cancer cells contacting specific extracellular matrix are able to turn chemotherapy into a proliferation-promoting signal, which contributes to drug resistance. For example, exposure to cisplatin induces proliferation of oral carcinoma cells while these cells adhere to carcinoma matrix through the function of integrin-*β*1, which transmits NF-*κ*B-dependent signal into the cells [65]. The angiogenesis-triggered resistance to chemotherapy was observed in non-small cell lung cancer cells as well [66]. A study using human non-small cell lung cancer cell lines and clinical specimens showed that higher expression of VEGF receptor-2, a vital angiogenesis-related receptor, in cancer cells was associated with the increased level of HIF-1*α* expression and resistance to platinum-based chemotherapy [66].

However, even though angiogenesis is the common leading cause of cancer progression, abruptly targeting the crucial contributing factor, VEGF, is still risky. The tumor microenvironment can serve as a clonal selection niche or compensatory substance providing source and thus make the resistance happen. Based on the assumption that tumor mass consists of multigenotypic population, the VEGF-targeting therapy may just play a selection pressure for selecting adapting tumor cells [67]. According to certain observations from human cancer studies, anti-VEGF therapy usually eventually results in regrowth of clonal populations with the characteristics of expressing higher compensatory factors such as VEGF, fibroblast growth factor (FGF), placental growth factor (PGF), and platelet-derived growth factor (PDGF) [67, 68]. Alternatively, the other tumor population with greater invasive ability would be favored after antiangiogenic therapy application, while the mechanisms remain under exploration [67, 69].

In addition, while the abnormal tumor vasculature is usually stagnant and functions poorly, anti-VEGF therapy reduced vessel size and tortuosity with more pericyte coverage of the remaining normalized vessels [70]. The growth factors pericytes secrete may facilitate vascular structure stabilization and normalization, which makes the tumors more adapting [67, 71]. Overall, the microenvironment may make the therapeutic outcome deviate from the original treatment purpose.

## 5. Limitations of Current* In Vivo* Model for Anticancer Drug Development

Since carcinogenesis is based on a complex individual genetic background, the outer environmental stimulation, and the delicate interplay between cancer cells, surrounding stromal cells, and infiltrating immune cells, the drug applying model should be chosen with caution to ensure adequate simulation of the clinical situation. Because* in vitro* cell culture system lacks the relevance of physiological clues for drug implementation, the investigation of curing efficacy through* in vivo* model is inevitable prior to the clinical application. Murine model has the advantages of easy manipulation, high fecundity, and close genetic background to human. Thus, the usage of murine model as preclinical* in vivo* trial is widely accepted. Mouse is a good animal model for assessing maximum tolerated dose of potential drugs (or drug toxicology). However, the mismatch between murine model and clinical cases in evaluating cancer progression activity or anticancer efficacy of drugs is usually acknowledged. This mismatch mainly comes from, as mentioned in several review literatures, the impossibility in simulating all detailed carcinogenic mechanisms in human, especially failure to create the same tumor microenvironment [72–75]. Even though many advanced murine models have been developed for addressing this issue, there are still much challenges waiting for breakthrough.

Xenografting human tumor to immune deficient mice (nude or severe combined immunodeficiency mice) has once been a keystone model for preclinical assessment of anticancer drug efficacy. However, the method is widely in debate nowadays. The major concern comes from the implanting site of tumor, which is usually located in subcutaneous region for easy observation of the drug's growing inhibition and tumor mass shrinking effect. Nonetheless, since subcutaneous region usually differs from tumor orthotropic architecture, it casts the doubt for further clinical treatment efficacy, because the drug penetrating ability or tumor evolving course may be quite different from the actual clinical situation. The previous survey about comparing the drug activity in phase I clinical trial with the corresponsive xenograft model showed that only 3.8% of drug with efficacy in xenograft murine model has positive effect in human [76]. The poor correlation in the drug effect between murine xenograft models and human beings also proposed other flaws of the murine model. Although the major purpose of clinical drug application is not only delimitating/eliminating the original tumor but also prohibiting cancer relapse/metastasis, the xenograft model almost never does well because it cannot simulate the participating process of immune system and the phenomena of tumor metastasis. Therefore, other alternative methods are being developed on the demand.

Orthotropic model and murine cancer syngeneic model are compromised choices. Orthotropically implanting human tumor to corresponsive tumor site on immune deficient mice takes the advantages to mimic the architecture of tumor primitive growth environment. Therefore, the behavior of rapid growth and distal metastasis can be evaluated. The similar histological features as the tumor original site also provide a more faithful microenvironment for assessing the targeting ability of the drug. By comparing the growth inhibiting effect of doxorubicin in mice implanting with human colon cancer cells ectopically or orthotopically, it is understandable that the effect can be quite different, from 80% inhibition in subcutaneous region to about 40% inhibition in orthotopic region [77]. Nonetheless, the necessity of using immune deficient mice is the major limitation for orthotopic model, as the contribution of immune cells to tumor progression is neglected. To retain the immune function, the murine cancer syngeneic model, using immune component mice inoculated with mice-originated cancer, seems to be more preferable. The great success of this model is the identification of potential antileukemia drug. The highly coherent correlations between drug fighting against intraperitoneally injected P388 or L1210 cell line (both are mice leukemia cell lines) and clinical application are persuasive. Subsequently, the murine subcutaneously injected B16 melanoma model and intravenously injected Lewis lung carcinoma model are developed in an attempt to screen the potential drug for treating the same type of cancer in human. However, the different cell characteristics between human tumors versus mice tumors finally drive the divergence of potential drug screening result. Actually, certain drug compounds which work in human-mouse xenograft model have shown negative response in the murine cancer syngeneic model. Taxol is one of such drugs that might be neglected if only relies on L1210 murine syngeneic test [78, 79].

Humanized mice and gene-modified mice are much advanced model for resolving the limitations that existed in the aforementioned models. Humanized mice allow the orthotropic implanting human tumor to be fostered in human-like immune condition through transplanting human stem cells or T cells from the donor of cancerous tissue to the immune deficient mice. Gene-modified mice can be used in much wide application. Through performing transgenic, knock-out, and knock-in technology to the mice, the mice can be modified to be more humanized via inserting human genome sequence in, or contributing to certain gene defect and representing autochthonous tumor models. All the above make the animal model more suitable for carrying out human cancer research. Nonetheless, certain technique limitations of these models are still waiting to conquer. Except much more capital and time investment to create these models, it is still uncertain whether the pharmaceutical efficacy in translation from mice to human can be highly improved [74, 75].

## 6. Future Drug Development regarding the Importance of Tumor Microenvironment

Since the tumor microenvironment contributes to many aspects of carcinogenesis and cancer progression and therefore offers promising treatment targets, any new inputs of tumor microenvironment may become the incentive of future anticancer drug development [50]. After further understanding the tumor microenvironment, it is undoubtedly that the concept for drug development required great revolution. Although the suppressive immunity is predominant in tumor microenvironment, immunomodulation should be used carefully as an anticancer treatment modality. Since the interaction between stromal and cancer cells is so essential for further tumor progression, the new signal molecules which play key roles for the crosstalk should be taken into account while seeking future treatment target. The environmental-mediated drug resistance points out that the drugs might turn into failure in a long timescale. To avoid the future resistance, the multitargeting drug or the “cocktail” drug application strategy may give a more favorable long-term outcome. Indeed, treatments targeting cancer cells as well as key components of the tumor microenvironment, as compared to chemotherapy alone, significantly improve the clinical outcomes [50]. To narrow down the gap between the experimental and clinical application of anticancer drugs, developing a preferable animal model seems inevitable.

In conclusion, because all the new directions for drug development are based on the wide knowledge of tumor microenvironment, understanding the mechanisms modulating tumor microenvironment may facilitate the design of a novel anticancer therapy and may obtain greater success in cancer eradication.
